# Molecular Mechanism Underlying Anti-Inflammatory and Anti-Allergic Activities of Phytochemicals: An Update

**DOI:** 10.3390/molecules18010322

**Published:** 2012-12-27

**Authors:** Yuva Bellik, Laïd Boukraâ, Hasan A. Alzahrani, Balkees A. Bakhotmah, Fatiha Abdellah, Si M. Hammoudi, Mokrane Iguer-Ouada

**Affiliations:** 1 Laboratory of Research on Local Animal Products, Ibn-Khaldoun University of Tiaret, Tiaret 14000, Algeria; E-Mails: bellik_youva@yahoo.fr (Y.B.); fatiha.abdellah@yahoo.fr (F.A.); mouh_hammoudi@yahoo.com (S.M.H.); 2 Faculty of Nature and Life Sciences, Abderrahmane Mira University, Béjaia 06000, Algeria; E-Mail: imokrane@gmail.com; 3 Mohammad Hussein Al Amoudi Chair for Diabetic Foot Research, King Abdulaziz University, Jeddah 21589, Saudi Arabia; E-Mails: haaz59@yahoo.com (H.A.A.); hbaab1961@yahoo.com (B.A.B.); 4 Department of Surgery, Faculty of Medicine, King Abdulaziz University, Jeddah 21589, Saudi Arabia; 5 Department of Nutrition Food Sciences, Arts and Design College, King Abdulaziz University, Jeddah 21589, Saudi Arabia

**Keywords:** phytochemicals, anti-inflammatory, anti-allergic, molecular mechanisms

## Abstract

The resort worldwide to edible medicinal plants for medical care has increased significantly during the last few years. Currently, there is a renewed interest in the search for new phytochemicals that could be developed as useful anti-inflammatory and anti-allergic agents to reduce the risk of many diseases. The activation of nuclear transcription factor-kappa B (NF-κB) has now been linked to a variety of inflammatory diseases, while data from numerous studies underline the importance of phytochemicals in inhibiting the pathway that activates this transcription factor. Moreover, the incidence of type I allergic disorders has been increasing worldwide, particularly, the hypersensitivity to food. Thus, a good number of plant products with anti-inflammatory and anti-allergic activity have been documented, but very few of these compounds have reached clinical use and there is scant scientific evidence that could explain their mode of action. Therefore, this paper intends to review the most salient recent reports on the anti-inflammatory and anti-allergic properties of phytochemicals and the molecular mechanisms underlying these properties.

## 1. Introduction

Plants have been the basis of many traditional medicine systems throughout the World for thousands of years and still remain as the main new source of structurally important chemical substances that lead to the development of innovative drugs [1,2]. The use of medicinal plants for the treatment of many diseases is associated with folk medicine from different parts of the World [3,4]. Nowadays, the search for new anti-inflammatory and anti-allergic agents from the huge array of medicinal plant resources is intensifying [5]. In fact, a variety of bioactive components have been shown to modulate inflammatory responses [6]. The inflammatory response is a critical protective reaction to irritation, injury, or infection, characterised by redness, heat, swelling, loss of function and pain [7]. Redness and heat result from an increase in blood flow, swelling is associated with increased vascular permeability, and pain is the consequence of activation and sensitisation of primary afferent nerve fibres [8].

The understanding of the cellular and molecular mechanisms involved in the inflammatory process has increased considerably in recent decades and this has permitted the discovery of many promising targets for the development of new drugs to treat chronic inflammatory diseases [8]. A great number of inflammatory mediators including kinins, platelet-activating factor (PAF), prostaglandins, leukotrienes, amines, purines, cytokines, chemokines and adhesion molecules, has been found to act on specific targets, leading to the local release of other mediators from leukocytes and the further attraction of leukocytes, such as neutrophils, to the site of inflammation [6].

The constant advent of new findings from immunohistochemical, biochemical, molecular and functional animal models, together with clinical trials, has greatly increased the interest in the study of the mechanisms that underlie the inflammatory process [8]. Recently, roles have been identified for several inflammatory cells and for a large number of inflammatory mediators in important pathologies not previously known to be linked to inflammation, such as Alzheimer’s disease and cardiovascular disorders including atherosclerosis, as well as cancer, reviewed in Akiyama *et al.* [9] and Libby *et al*. [10].

Natural products have long been, over the years, contributed to the development of modern therapeutic drugs [11]. Evidence exists that drugs derived from natural products can modulate various inflammatory mediators (arachidonic acid metabolites, peptides, cytokines, excitatory amino acids, *etc*.), the production and/or action of second messengers (cGMP, cAMP, protein kinases, and calcium), the expression of transcription factors such as AP-1, NF-κB, and proto-oncogenes (*c-jun*, *c-fos*, and *c-myc*), and the expression of key pro-inflammatory molecules such as inducible NO synthase (iNOS), cyclooxygenase (COX-2), cytokines (IL-1β, TNF-α), neuropeptides and proteases [6,7,8].

In parallel, the allergic process has an important inflammatory component in which mast cell activation and degranulation are the first phenomena observed. During this process, mast cells release several inflammatory mediators including histamine (5-HT), platelet aggregating factor (PAF), leukotrienes, and a variety of cytokines [12,13]. Hypersensitivity type I, an allergic reaction, is an IgE mediated immune response, resulting in histamine secretion from mast cells and blood basophils. The early phase reaction of allergy occurs within minutes after allergen exposure, whereas the late phase reaction occurs hours later and involves in cytokines secretion such as TNF-α and IL-4 [14].

The discovery of drugs that can be used for the treatment of inflammatory and allergic diseases is important in human health. Drug discovery from plants involves a multidisciplinary approach combining botanical, ethnobotanical, phytochemical and biological techniques [2]. Several natural product drugs of plant origin are in clinical use and some are undergoing Phase II and Phase III clinical trials [2,3,4,5]. This review highlights the current patents about the potential benefits and effectiveness of phytochemicals that have shown experimental or clinical anti-inflammatory or anti-allergic activities, the possible mechanism of action and their therapeutic value.

## 2. Major Classes of Phytochemicals

**Figure 1:**
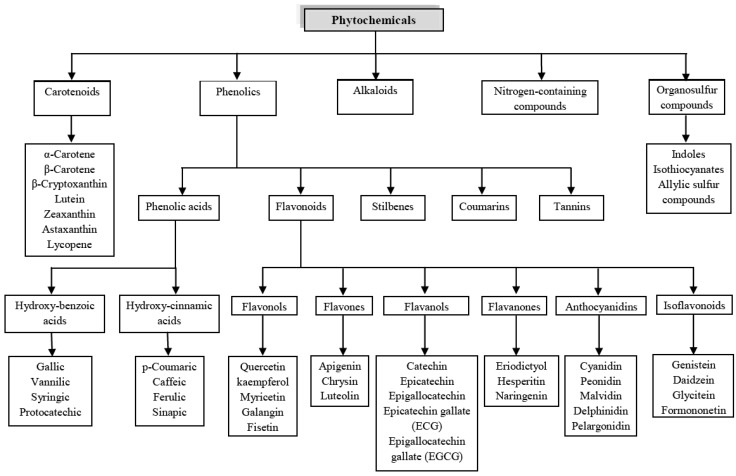
Classification of dietary phytochemicals [16].

Plants are rich in a wide variety of secondary metabolites, the great majority of which do not appear to participate directly in growth and development [15]. Based on their biosynthetic origins, phytochemicals can be classified as carotenoids, phenolics, alkaloids, nitrogen-containing compounds, and organosulfur compounds. Interestingly, an important classification has been depicted by Liu [16] gathering nearly most of dietary phytochemical classes and the structures of their main chemically relevant components (Figure 1).

Phytochemicals, although noted for the complexity of their chemical structures and biosynthetic pathways, they have been widely perceived as biologically insignificant and have historically received little attention from most plant biologists. Organic chemists, however, have long been interested in these novel phytochemicals and have investigated their chemical properties extensively since the 1850s [15]. At present numerous studies have established that the phytochemical content of plants contributes to their protective effects against acute, chronic, and degenerative diseases [17,18,19].

## 3. Molecular Mechanism Underlying Phytochemicals

### 3.1. Inflammation

Wide ranges of phytoconstituents were responsible for anti-inflammatory activity including phenolics, alkaloids, and terpenoids [19]. However, efforts have focused on a class of compounds to elucidate the mechanisms of action of herbs, characterize and establish their potential utility as therapeutic agents in the treatment of inflammatory diseases.

Several mechanisms of action have been proposed to explain the anti-inflammatory actions of phytoconstituents, it consist broadly in: (1) Antioxidative and radical scavenging activities; (2) Modulation of cellular activities of inflammation-related cells (mast cells, macrophages, lymphocytes, and neutrophils); (3) Modulation of proinflammatory enzyme activities such as phospholipase A_2_ (PLA_2_), cyclooxygenase (COX), and lipoxygenase (LOX) and the nitric oxide (NO) producing enzyme, nitric oxide synthase (NOS); (4) Modulation of the production of other proinflammatory molecules; (5) Modulation of proinflammatory gene expression.

**Figure 2:**
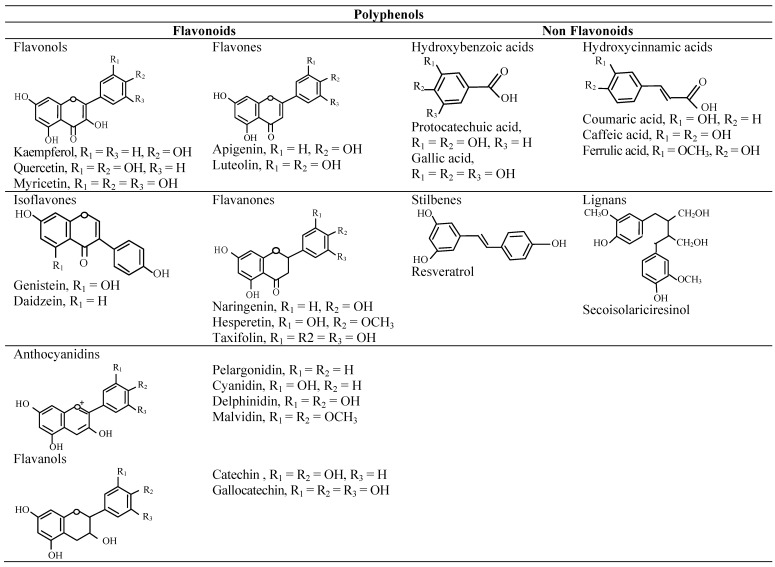
Chemical structures of polyphenols. Modified from Vauzour [20].

**Figure 3:**
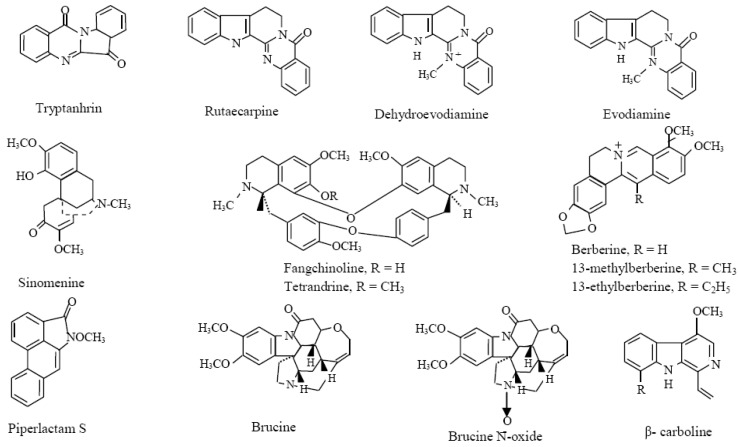
Chemical structures of alkaloids. Adapted from Gautam and Jachak [7].

**Figure 4:**
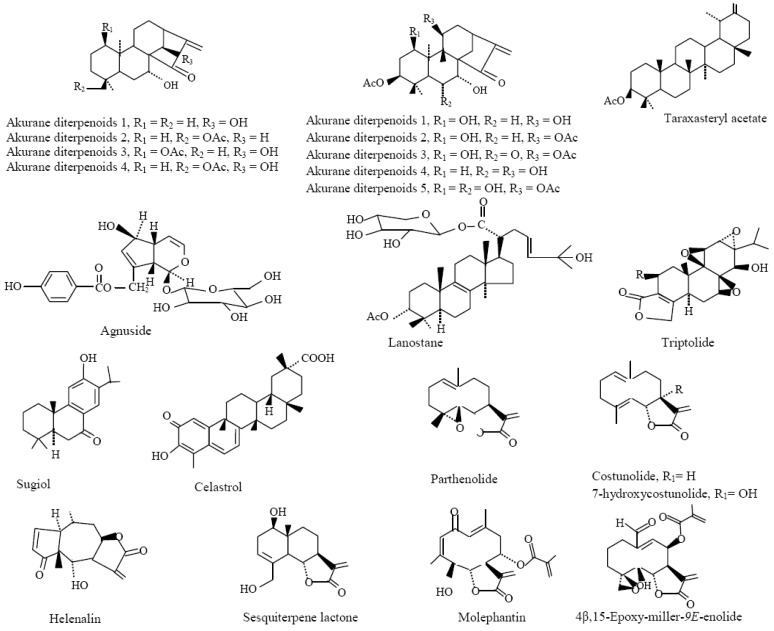
Chemical structures of terpenoids. Adapted from Gautam and Jachak [7].

The Table 1 and Table 2 summarize the most studied and well-known phytochemicals including polyphenols (Figure 2), alkaloids (Figure 3), and terpenes (Figure 4) compounds with anti-inflammatory activities and their cellular and molecular mechanism. It should be noted that several other reports demonstrating the similar results are not represented here. 

**Table 1:** Anti-inflammatory activities of phytochemicals.

Target pathway	Effects	Compounds	Mechanism of action	References
**Antioxidative and radical scavenging activities**	*Promoting antioxidant enzymes activity*	Quercetin, resveratrol, curcumin, hydroxytyrosol, catechin, luteolin	Increasing the activity of superoxide dismutase (SOD), catalase (CAT), glutathione peroxidase (GPx), glutathione reductase (GR), glutathione *S*-transferase (GST), γ-glutamylcysteine synthetase (γ-GCS) NADPH:quinone oxidoreductase-1 (NQO1) and heat shock proteins 70 (HSP70) expression	[21,22,23,24,25,26,27,28,29,30]
	*Inhibiting pro-oxidant enzymes activity*	Epigallocatechin, ECG, EGCG	Inhibiting lipoxygenase and cyclooxygenase	[31]
		Typheramide, alfrutamide, (−)-epicatechin, procyanidin	Inhibiting the activities of 5- lipoxygenase, 12-lipoxygenase and 15-lipoxygenase	[32,33]
		Curcumin, resveratrol, lupeol	Decreasing the activity of iNOS and myeloperoxidase (MPO) level	[24,30,34]
		Ellagic acid gallic, acid corilagin, luteolin	Inhibiting tyrosinase and xanthine oxidase	[35,36]
		Resveratrol	Inhibiting *O*-acetyltransferase and sulfotransferase activities	[37]
	*Prevent free radical attacks*	Epicatechin, rutin, mannitol	Scavenging hydroxyl radical (OH^.^)	[38]
		Ellagic acid gallic, acid corilagin, luteolin, β-carotene, tetrandrine	Scavenging superoxide radical (O_2_^.^)	[35,36,39,40]
		Quercetin, curcumin, lycopene	Decreasing MDA and lipoperoxidation	[22,30,41]
	*Enhancing endogenous antioxidant molecules*	Quercetin, resveratrol, catechin, proanthocyanidin B4, β-carotene	Elevating cellular GSH content	[21,24,26,42]
**Modulation of cellular activities of inflammation-related cells**	*Inhibition of enzymes involved in signaling transduction and cell activation processes (T cell, B lymphocyte) or cytokine production*	Genistein	Inhibition of tyrosine protein kinaseinducing anti-proliferative effects on T cell, reducing IL-2 secretion and IL-2R expression	[43,44]
		Quercetin, kaempferol, apigenin, chrysin, luteolin	Inhibition of tyrosine protein kinaseinducing anti-proliferative effects on M-CSF-activated macrophages	[45]
	*Inhibition of arachidonic acid release from membranes (degranulation)*	Quercetin	Inhibiting lysosomal enzyme release from stimulated neutrophil (elastase, β-glucuronidase)	[46,47,48]
			Impairing lysosomal enzyme release from polymorphonuclear leukocytes	[47,49,50]
		Rutin	Reducing the polymorphonuclear neutrophils chemotaxis to FMLP	[51]
**Modulation of arachidonic acid (AA) related enzymes**	*Inhibition of arachidonic acid metabolism*	Quercetin, kaempferol, myricetin, hesperetin, naringenin, quercetagetin, kaempferol-3-galactoside, scutellarein, ochnaflavone, amentoflavone, ginkgetin, morelloflavone, bilobetin, triptolide, papyriflavonol A	Inhibition of PLA2 activity	[50,51,52,53,54,55,56,57,58,59]
	*Inhibition of proinflammatory enzymes (COX, LOX and NOS) from different sources*	Luteolin, 3',4'-dihydroxyflavone, galangin, morin, apigenein, chrysin, quercetin, myricetin, morusin, kuwanon C, sanggenon D, broussoaurone A, cycloheterophyllin, broussochalcone A broussoflavonol F, catechin, EGCG, resveratrol, xanthomicrol, cirsiliol, hypolaetin, diosmetin, tectorigenin, kuraridin, kurarinone, sophoraflavanone G, morusin, sanggenon B, kazinol B, rutaecarpine, 1,2-di-*O*-α-linolenoyl-3-*O*-β-galactopyranosyl-*sn*-glycerol (dlGG), curcumin, 4'-Me-gallocatechin, lonchocarpol A, tomentosanol D, catechins, catechins gallate	Inhibited COX activity	[6,58,60,61,62,63,64,65,66,67,68,69,70,71,72,73,74]
		Sophoraflavanone G, kenusanone A, kuraridin, papyriflavonol A, sanggenon B, sanggenon D, boswellic acid, diphyllin acetylapioside	Inhibited 5-LOX activity	[69,75,76,77]
		Quercetin, kaempferol, fisetin, quercetagetin-7-*O*-glucoside, hibifolin, hypolaetin, sideritoflavone, 5,6,7-trihydroxyflavone (baicalein)	Inhibited 12-LOX activity	[6,78]
		Kaempferol, quercetin, myricetin, morin, cirsiliol, artonins	Inhibited 5-LOX and 12-LOX activity	[79,80,81,82]
		Quercetin	Inhibited eNOS activity	[83]
**Modulation of the production of other proinflammatory molecules**	*Inhibition of proinflammatory cytokines from different sources*	Formononetin	Inhibited iNOS activity	[84]
		Genistein, apigenin, quercetin, morin, wogonin, soyisoflavones, daidzein, glycitein, dlGG, paeonol	Inhibited NO production	[71,85,86,87,88,89]
		Genistein, quercetin, wogonin, baicalein, luteolin, nobiletin, paeonol, chlorogenic acid, hematein, aucubin, catalposide, tetrandrine, fangchinoline, colchicines, piperlactam S	Inhibited cytokine production : IL-1β, IL-6, TNF-α	[89,90,91,92,93,94,95,96,97,98,99,100,101]
		Curcumin, amoradicin, genistein, silybin, quercetin, wogonin, rutin, luteolin, eriodictyol, hesperitin, EGCG, geraniin, corilagin, pinoresinol, woorenoside, lariciresinol glycoside, terpinen-4-ol, physalin B, triptolide, lupeol, [6]-shogaol, vitamin D, cepharanthine, fangchinoline, adenosine	Inhibited TNF-α production	[34,98,102,103,104,105,106,107,108,109,110,111,112,113,114,115,116,117,118,119,120,121,122,123]
		Apigenin, wogonin, bacalein	Inhibited IL-6 and IL-8 production	[124,125]
		Genistein, ilicic acid, inuviscolide acid, tryptanthrin	Inhibited LTB_4_ production	[126,127,128]
		Saikosaponins, masticaienonic acid, masticadienolic acid, morolic acid	Reducing LTC_4_ production	[128,129,130,131]
		Chrysin, flavone, galangin, kaempferol, quercetin, salidroside, syringin, phillyrin, coniferin, tryptanthrin	Inhibited TXB_2_ production	[79,128,132]
		Lupeol, paeonol, quercetin, salidroside, syringin, phillyrin, tectorigenin, tectoridin, platycodin D, β-turmerone, ar-turmerone, rutaecarpine	Inhibited PGE2 production	[34,89,105,132,133,134,135,136]
**Modulation of proinflammatory gene expression**	*Inhibition of the expression of various inflammation-related proteins/enzymes, by suppressing activation of transcription factors such as NF-κB and AP-1*	Baicalein, oroxylin A, baicalin, skullcapflavone II	Inhibited eotaxin production	[137]
		Rutin, bilobetin, ginkgetin, isoginkgetin, ochnaflavone, morusin, kuwanon C, kazinol B, sanggenon B and D, echinoisoflavanone, wogonin, apigenin, kaempferol, genistein, chrysin, luteolin, quercetin, myricetin, flavone, tectorigenin, nobiletin, oroxylin A, galangin, EGCG, isoliquiritigenin, silymarin, curcumin, flavones, daidzein, glycitein, isorhamnetin, naringenin, pelargonidin, soyisoflavones, wogonin, resveratrol, triptolide, lupeol, butyrate, zeaxanthin, β-carotene	Inhibited iNOS expression	[56,84,87,138,139,140,141,142,143,144,145,146,147,148,149,150,151,152,153,154,155,156,157]
		Bilobetin, ginkgetin, paeonol, tectorigenin, tectoridin, platycodin D, apigenin, genistein, kaempferol, quercetin, myricetin, nobiletin, rhamnetin, eriodictyol, luteolin, fisetin, phloretin, wogonin, galangin, oroxylin A, lupeol, isoliquiritigenin, amentoflavone, butyrate, ursolic acid, iridoid, pendunculariside, agnuside, ferulic acid, [6]-Gingerol, resveratrol, EGCG	Inhibited COX-2 expression	[56,89,133,134,140,141,142,143,147,154,158,159,160,161,162,163,164,165,166,167,168,169,170,171,172]
		Lycopene, dlGG, wogonin, genistein, apigenin, kaempferol, myricetin, oroxylin, silymarin, β-carotene, resveratrol, quercetin, avicins, parthenolide, chlorogenic acid, triptolide, capsaicin, butyrate, luteolin, curcumin	Inhibition of NF-κB activation	[41,71,87,90,140,142,145,148,157,171,173,174,175,176,177,178,179,180,181]
		Hematein, casearinols A and B, casearinones A and B, colchicine	Inhibited the expression of ICAM-1 and VCAM-1 on the surface of different cells	[95,182,183]

**Table 2:** Phytochemicals with anti-inflammatory effects and their clinical efficiencies.

Herbal formulation/Compound	Indication	Clinical efficiency	References
Curcumin	Antirheumatic	- Exerted an antirheumatic activity comparable to that of phenylbutazone	[184]
Active constituents of honeysuckle (*Lonicera japonica)* stem	Anti-inflammatory and analgesic effect	- Prevented croton oil induced-mice ear edema - Inhibited arachidonic acid-induced mice ear edema - Inhibited writhing reaction in mice induced by acetic acid	[185]
Cocoa extracts containing polyphenols enriched with procyanidins	COX and/or lipooxygenase (LOX) modulators, NO or NO-synthase modulators, as non-steroidal anti-inflammatory agents, platelet aggregation modulators, antioxidants, inhibitors of oxidative DNA damage and DNA topoisomerase II inhibitor	- Inhibition of the COX-1 and COX-2 activities from ram seminal vesicle and sheep placenta - Inhibition of DNA topoisomerase II - Effect on LPS-induced nitrite production by γ-interferon-primed monocytes/macrophages - Effective on cancer cells such as: KB Nasopharyngeal/HeLa cell line, HCT-116 cell line, ACHN renal cell line, A-549 lung cell line, SK-5 melanoma cell line, MCF-7 breast cell line, CCRF-CEM T-cell leukemia cell line, MDA MB231 breast cell line, PC-3 prostate cancer cell line, Hela cervical cancer cell line, SKBR-3 breast cancer cell line, CRFK normal kidney cell line, MDCK normal kidney line, canine GH normal kidney cell line	[186]
Composition comprising: *Ajuga turkestanica*, *Panax quinquefolius*, *Rhodiola rosea* root, *Glycyrrhiza glabra*, *Morinda citrifolia* fruit, *Uncaria tomentosa* inner bark, *Capsicum frutescens*, chondroitin sulfate, *Curcuma longa*, *Dioscorea villosa*, glucosamine sulfate, *Harpagophytum procumbens* and *Tribulus terrestris*	Treating arthritis and its symptoms, rheumatoid arthritis and osteoarthritis as well as any inflammatory condition of the joints and their symptoms, pain swelling, heat, redness and limitation of movement	- The formulation is revealed to be an excellent alternative for the handling of osteoarthritic patients with femoropatteral knee, chondromalacia and meniscopathy	[187]
Synergistic mixture of standardized *Boswellia serrata* extract, glucosamine salts, and curcuminoids. The composition optionally containing bromelain, chondroitin, methylsulphonylmethane, resveratrol, extracts of white willow and ginger, and quercetin.	Treating and controlling inflammatory diseases, preventing and curing cancer	- Protective effect on adjuvant induced arthritis in winstar albino rats	[188]
Extracts of *Vitex leucoxylon* and its constituents: corosolic acid, agnuside and 6-*O*-caffeoylarbutin	Inflammatory diseases, diabetic conditions, liver disorders and free radical mediated diseases	- Anti-inflammatory activity by preventing carrageenin induced paw edema in albino wistar rats	[189]
Carotenoids, and xanthophyll carotenoids, or analogs or derivatives of astaxanthin, lutein, zeaxanthin, lycoxanthin, lycophyll, or lycopene	Reduce the adverse side effects associated with administration of COX-2 selective inhibitor drugs. Reduce peroxidation of low density lipoprotein (LDL) and other lipids in the serum and plasma cell membranes, and reduce the incidence of deleterious clinical cardiovascular events of subjects undergoing COX-2 selective inhibitor drug therapy	- Inhibition of the superoxide anion - Decrease of the lag time for LDL conjugated diene formation and increase of the levels of thiobarbituric-acid-reactive-substances (TBARS) - Increase of isoprostane formation from lipid vesicles enriched with arachidonic acid - Increase in electron density associated with the upper hydrocarbon core of the membrane	[190]
Two herbal compositions. The first composition comprises Radix Clematidis, Radix Angelicae Pubescentis, Rhizoma et Radix Notopterygii, Radix Saposhnikoviae, and Radix Gentianae Macrophyllae. The second composition comprises Rhizoma Chuanxiong, Radix Angelicae Sinensis, Cortex Eucommiae, and Radix Achyranthis Bidentataeas	Preventive and therapeutic effects on alleviating symptoms associated with inflammatory and rheumatic diseases	- Effective on patients with rheumatoid arthritis and lack severe side effects	[191]
[5-hydroxy-7-methoxy-2-(4'-methoxyphenyl}-4-*oxo*-4H-chromen-8-yl] sulfonic acid monoester obtainable by extraction of plant material selected from *Sidastrum acuminatum*, *Sidastrum burrerense*, *Sidastrum* E.G. Baker, *Sidastrum kicranthum*, *Sidastrum lodiegense*, *Sidastrum multiflorum*, *Sidastrum micranthum*, *Sidastrum paniculatum*, *Sidastrum strictum*, *Sidastrum tehuacanum* or *Sidastrum quinquenervium*	Inhibits the arachidonic acid cascade	- Antiinflammatory properties keratinocyte monolayer PGE 2 model - Induction of gene expression by transglutaminase which plays a crucial role in the formation of jacket surrounding the keratinocytes	[192]
Oil-soluble licorice extract	Inhibitory effect on: hyaluronidase activity, hexosaminidase release, platelet aggregation, and phospholipase A2 activity, and which is suitably used especially as an external preparation for skin	- Inhibitory effect on hyaluronidase activity of bovine testis - Inhibitory effect on hexosaminidase release from rat basophilic leukemia cells - Inhibitory effect on rabbit platelet aggregation - Inhibitory effect on phospholipase A2 activity of rat leukemia cells	[193]
Extracts or fractions of *Aphanamixis polystachya*	Diseases mediated by 5-lipoxygenase enzyme	- Inhibition of 5-Lipoxygenase activity - Inhibition of tyrosinase activity - Anti-oxidant and anti-inflammatory activities by acting on the following target molecules : nitrite, TNF-α, IL-1β and the levels of lipid peroxidation and glutathoine in the liver of Freund complete adjuvant induced arthritis model of Sprague Dawley rats	[194]
Extracts and fractions from *Hypericum* *gentianoides*	Inhibition of inflammation, PGE2-mediated disease, disorder or condition, a COX-mediated disease, disorder or condition, or an infection of HIV	- Reduced LPS-induced COX-2 enzyme in RAW 264.7 macrophages - Reduced LPS-induced PGE2 in RAW 264.7 macrophages - Reduced HIV infection *in vitro*	[195]
Compositions containing one or more of a flavone or flavonoid glycoside a non-bovine heavily sulfated proteoglycan, an unrefined olive kernel extract, a hexosamine sulfate, a histamine-1 and histamine-3 receptor agonist, an antagonist of CRH, a long-chain unsaturated fatty acid, a phospholipid, Krill oil, a polyamine, glutiramer acetate and interferon	Treatment of inflammatory conditions. Inhibitors of mast cell activation and secretion in the brain as in multiple sclerosis	- Increased the absorption of a proteoglycan (chondroitin sulfate) from the intestine into the general circulation in Sprague-Dawley rats	[196]
Berry extract containing stable anthocyanin	Treating inflammation, oxidative damage, or cancer	- Inhibition of proliferation of HT-29 human colorectal cancer cells - Ihibition of IL-12 release from murine dendritic cells	[197]
Free-B-Ring flavonoids from *Scutellaria baicalensis*	Treatment of COX-2 mediated diseases and conditions	- Inhibition of COX-1 of THP-1 cells and COX-2 of HOSC cells	[198]

The inflammatory process can be initiated by various inflammatory stimuli including viruses, chemicals, and reactive oxygen/nitrogen species, which subsequently increases the synthesis and secretion of proinflammatory cytokines. Moreover, the unchecked activation of NF-κB/AP-1 and the production of TNF-α signaling have provided compelling evidence about the critical role for these factors in coupling inflammation and many chronic diseases. Phytochemicals have been shown to modulate various points in these inflammatory processes [6]. These modulations serve as controlling points where the amplification of the inflammatory processes can be disconnected and thereby reduce subsequent diseases risk.

### 3.2. Allergy

The allergic process has an important inflammatory component. Hypersensitivity reactions can be divided into four types:

Type I: Called immediate or anaphylactic hypersensitivity mediated by IgE. Mast cells and basophils play a central role in immediate allergic inflammation through releasing chemical mediators such as histamine and cysteinyl leukotrienes, cytokines and chemokines. The reaction may involve skin (eczema), eyes (conjunctivitis), nasopharynx (rhinitis), bronchopulmonary tissues (asthma) and gastrointestinal tract (gastroenteritis).

Type II: Known as antibody-mdiated cytotoxicity mediated by antibodies of the IgM or IgG classes and complement. Antibodies directed against cell surface antigens causes cell damage such as hemolytic disease of the newborn (Rh disease) and myasthenia gravis (MG).

Type III: Known as immune complex hypersensitivity mediated by IgG or IgM classes. The reaction may be general (serum sickness) or may involve individual organs including skin (systemic lupus erythematosus), joints (rheumatoid arthritis) or other organs.

Type IV: Known as cell mediated or delayed type hypersensitivities. These reactions are mediated by CD4+T cells, and involved in the pathogenesis of many autoimmune diseases (multiple sclerosis). Another form of delayed hypersensitivity is contact dermatitis (poison ivy).

Therapeutic intervention in allergic disease has thus commonly focused on suppressing IgE production and blocking the action of histamine, thus regulating the expression and/or release of cytokines, chemokines, adhesion molecules, and or/inflammatory mediators. Below (Table 3 and Table 4) are summarized some of the most studied and well-known phytochemicals with anti-allergic effects and their mode of action. Here, too, several other reports demonstrating the similar results are not represented.

**Table 3:** Anti-allergic activities of phytochemicals.

Target pathway	Effects	Compounds	Mechanism of action	Ref.
Effect on IgE-mediated Hypersensitivity (Type I)	*Inhibition of chemical mediator release and cytokine production by mast, basophil or T cells*	Luteolin, quercetin, baicalein	Inhibited the release of histamine, leukotrienes and prostaglandin D_2_ Inhibited IgE-mediated TNF-α and IL-6 production	[199]
		Luteolin, quercetin, baicalein, apigenin	Inhibited the p44/42 MAPK phosphorylation in response to crosslinkage of FcεRI	[200]
		Tetrandrine	Suppression of prostaglandin and leukotriene generation	[201]
		Coixol, pseudoephedrine, mallotophilippen A and B	Inhibited the release of histamine	[202,203,204]
		Apigenin, luteolin, 3.6-dihydroxy flavones, fisetin, kaempferol, quercetin, myricetin	Inhibition of the hexosaminidase release Suppression of cysteinyl leukotriene synthesis	[205]
		Flavone, quercetin	Inhibition of transport ATPase in histamine secretion	[206,207]
		Isoquercitrin	Inhibited carbachol and leukotriene D_4_ production	[208]
		Cirsiliol (3',4',5-trihydroxy-6,7-dimethoxy flavone)	Suppressed cysteinyl leukotrienes release	[80]
		Ayanin, luteolin, apigenin, diosmetin, fisetin, ombuin, quercetin, kaempferol (*other compounds see* Table 1)	Suppression of IL-4 synthesis (*other cytokines see Table 1*)	[209]
	*Inhibition of signal transduction and gene expression in mast, basophil or T cells* Preventing allergic asthma	Mallotophilippen A and B (*other compounds see* Table 1)	Inhibited iNOS gene expression (*other enzymes see Table 1*)	[204]
		Luteolin, apigenin, fisetin	Suppressed CD_40_ ligand expression	[209,210]
		Nobiletin	Reduced eotaxin expression	[211]
		Luteolin, apigenin, fisetin	Inhibited AP-1 and NFAT activation	[210]
		Dietary polyphenols	Interfer with activated T-helper 2	[212]
		Quercetin, provinol, flavin-7	Anti-inflammatory effects in experimental allergic asthma	[213,214,215]
Effect on cell-mediated hypersensitivity (type IV)	*Preventing contact dermatitis*	Polyphenol (extract from the bark of *Acacia mearnsii*)	Inhibited itching in atopic dermatitis by preventing the skin from drying	[216]
		Polyphenols and anthocyanins derived from *Vaccinium uliginosum* L	Improve atopic dermatitis disease in mice by reducing the Th2/Th1 ratio, IL-4 and IL-13 (as Th2 cytokines), IFN-γ, and IL-12 (as a Th1 cytokine) in spleens Decreased gene expression, such as IL-4, IL-5, CCR3, eotaxin-1, IL-12, IFN-γ, MCP-1, and IL-17, and suppressed Th 17	[217]
Attenuating autoimmune disorders	*Improving multiple sclerosis (MS) disease*	Dietary polyphenols, carotenoids, curcumin	Inhibited neuroinflammation in MS Inhibited the differentiation and expansion of Th17 cells in circulation induced by inflammatory cascade; Enhanced the expression of ZO-1; Down-regulated expression of CXC chemokines and receptor; Decreased Th17 cells to transmigrate across the blood brain barrier and the inhibition of autoreactive T cells transmigration can reduce neuroinflammation; Blocked IL17 and others, which lead to centtral system nervous tissue destruction in MS	[218,219,220]

**Table 4:** Phytochemicals with anti-allergic effects and their clinical efficiencies.

Herbal formulations/Compounds	Indication	Clinical efficiency	Ref.
Seeds of *Cucurbita moschata* and flowers of *Carthamus tinctorius* and at least one crude drug selected from *Plantago asiatica*, *Lonicera japonica*, *Glycyrrhiza uralensis*, *Coix lachrymal*-*jobi* var. *ma*-*yuen*, *Zingiber officinale*, *Curcuma longa*, *Curcuma zedoaria* and *Artemisia argyi.*	Prevention or therapy of pollen allergy, allergic rhinitis, atopic dermatitis, asthma or urticaria	Animal trials: Inhibiting the production of total IgE antibodies in the blood of mice sensitized with cedar pollen Human trials: Therapeutic effects on patients suffering from cedar pollen allergy	[221]
Formulation(s) comprises of *Tinospora cardifolia*, *Piper longum*, *Albizia lebbeck* and *Curcuma amada*	Treatment of allergy	Decreased the histamine release (mast cell degranulation) in rats-Reduced lipid peroxidation and superoxide dismutase activity, and increased catalase activity in tissues (liver, kidney and heart) rats	[222]
The composition comprises at least one of the following ingredients: luteolin from Perilla leaf or seed, Cinnamon, Kiwi, Picao preto, Hesperidin, Acerola cherry, Guaco, Holy Basil, Kakadu, Solamum, Rosmarinic acid, Tinospora and Aframomum	Inhibits and/or mitigates an allergic response	Inhibition of the IgE secretion by U266 human myeloma cells-Reduction of the IgE receptor expression by RBL-2H3 cells-Inhibiting or preventing the release of mediators such as histamine, PGD 2 and LTC4 by RBL-2H3 cells	[223]
Flavonoid and/or a flavonoid derivative (Troxerutin or Veneruton^®^)	Treating symptoms of common cold, allergic rhinitis and infections relating to the respiratory tract	Showed success results on different patients suffering from common cold symptoms-Reduced the symptom score after treatment of patients suffering from allergic airway conditions	[224]
Kaempferol, apigenin	Treatment of contact dermatis	Inhibited iNOS induction produced in contact dermatitis	[225]
Dehydrocorydaline	Treatment of hypersensivities reactions	Inhibited the induction phase of picryl chloride-induced contact dermatitisin mice	[226]

Despite the promising use of plant products for medicinal purposes for the evidences discussed above, it is worth noting that many of the dietary phytochemicals or natural products are not without cytotoxic effect and can originate various allergic reactions. The well known allergenic phytoconstituents are sesquiterpene lactones and furanocoumarins. Many of plants containing sesquiterpene lactones cause allergic contact dermatitis and effective treatments are scarce. Other natural products such as flavonoids [227], alkaloids [228,229], and terpenoids [230,231] can also cause allergic reactions. Phenolics such as: anethol, atranorin, catechols, cinnamon, cinnamic derivatives, benzoic acid, curcumin, eugenol, isoeugenol, litreol, ginkgolic acid, resorcinols, oak moss resin, tertiaery-butylhydroquinone, urushiol, usnic acid. Alkaloids such as: atropine, pilocarpine, quinine, thebaine, codeine, and terpenoids such as: abietic acid, alantolactone, artesunate, asiaticoside, asiatic and madecassic acids, carvone, citral, β-cyclocostunolide, dehydroabietic acid, eucalyptol, farnesol, geraniol, limonene, α-pinene, phellandrene, linalool, menthol, myrrh, parthenolide, polygodial, sesquiterpenes, sesquiterpenes, thymol (reviewed in Rios *et al.* [232]). While flavonoids are only weakly antigenic and usually do not induce immune reactions after consumption or therapeutic application, antibodies against flavonoids have been found in human blood [227]. Adverse side effects of polyphenol intake on cardiovascular diseases have been also reported. A high consumption of polyphenol (2 g chlorogenic acid per day during 1 week) significantly increased homocysteinemia [233,234]. The consumption of tea has been associated with a higher bone mineral density [235]. A recent randomized crossover trial [236] revealed that moderate consumption of red wine reduced erythrocyte superoxide dismutase activity. Another randomized double-blind, placebo-controlled trial showed that the combination of vitamin C and grape-seed polyphenols increases blood pressure [237].

## 4. Conclusions

Phytochemicals show both anti-inflammatory and anti-allergic activities *in vitro* and *in vivo*. Several cellular action mechanisms are proposed to explain their mode of action. Any single mechanism could not explain all of their *in vivo* activities. They probably have multiple cellular mechanisms acting on multiple sites of cellular machinery. The continual efforts will provide new insight into the anti-inflammatory and anti-allergic activities of phytochemicals, and eventually lead to development of a new class of anti-inflammatory and anti-allergic agents. However, the concern and difficulties related to the investigation of herbal medicines have precluded the financial incentives that could be provided to pharmaceutical industries. As a function of such difficulties, few herbal drugs have been studied adequately and well-controlled double-blind clinical trials to prove their safety and efficacy have been lacking. The trend today, especially in an industrial setting, is to seek bioactive compounds from plants that will serve as lead compounds for synthetic or semisynthetic development, and knowledge of the main pharmacologically active plant compounds is an essential requirement to standardize procedures for obtaining herbal remedies in order to replace crude products with modern pharmacological formulations.
